# Postoperative Period of Myocardial Revascularization Surgery: Retrospective Cohort Study of a Single Center

**DOI:** 10.21470/1678-9741-2022-0332

**Published:** 2023-07-18

**Authors:** Ana Carolina Longui Macedo, Antônio Luís Eiras Falcão, Luiz Claudio Martins, Orlando Petrucci Junior, Marcos Mello Moreira

**Affiliations:** 1 Postgraduate Program in Sciences of Surgery, Department of Internal Medicine, Faculdade de Ciências Médicas, Universidade Estadual de Campinas, Campinas, São Paulo, Brazil; 2 Intensive Care Unit, Department of Sciences of Surgery, Faculdade de Ciências Médicas, Hospital de Clínicas da Universidade Estudual de Campinas, Campinas, São Paulo, Brazil; 3 Discipline of Internal Medicine and Semiology, Postgraduate Program in Sciences of Surgery, Department of Internal Medicine, Faculdade de Ciências Médicas, Universidade Estadual de Campinas, Campinas, São Paulo, Brazil; 4 Cardiac Surgery Division, Faculdade de Ciências Médicas, Universidade Estadual de Campinas, Campinas, São Paulo, Brazil; 5 Discipline of Pneumology, Postgraduate Program in Sciences of Surgery, Department of Internal Medicine, Faculdade de Ciências Médicas, Universidade Estadual de Campinas, Campinas, São Paulo, Brazil

**Keywords:** Coronary Artery Byspass, Off-Pump, Artifical Respiration, Body Mass Index, Ventilator Associated Pneumonia, Length of Stay, Renal Dialysis, Mortality

## Abstract

**Introduction:**

Risk factors and postoperative complications can worsen the condition of patients undergoing coronary artery bypass grafting; some of these factors and complications are closely related to mortality rate.

**Objective:**

To describe clinical factors and outcomes related to mortality of patients undergoing coronary artery bypass grafting and on invasive mechanical ventilation.

**Methods:**

This is a single-center retrospective data analysis of patients who underwent coronary artery bypass grafting on invasive mechanical ventilation between 2013 and 2019. Data regarding clinical characteristics, postoperative complications, intensive care unit and mechanical ventilation time, and their relationship with mortality were analyzed.

**Results:**

Four hundred seventy-two patients who underwent coronary artery bypass grafting entered the study. Their mean age was 62.3 years, and mean body mass index was 27.3. The mortality rate was 4%. Fifty percent of the patients who had ventilator-associated pneumonia died. Considering the patients who underwent hemotherapy and hemodialysis, 20% and 33% died, respectively. Days of intensive care unit stay and high Acute Physiology and Chronic Health Evaluation score and Simplified Acute Physiology Score were significantly related to death.

**Conclusion:**

Factors and clinical conditions such as the patients’ age, associated comorbidities, the occurrence of ventilator-associated pneumonia, length of stay in the intensive care unit, and mechanical ventilation time are related to higher mortality in patients undergoing coronary artery bypass grafting.

## INTRODUCTION

**Table:** 

Abbreviations, Acronyms & Symbols	
AMI	= Acute myocardial infarction
APACHE	= Acute Physiology and Chronic Health Evaluation
BMI	= Body mass index
CABG	= Coronary artery bypass grafting
DM	= Diabetes mellitus
EuroSCORE	= European System for Cardiac Operative Risk Evaluation
ICU	= Intensive care unit
MV	= Mechanical ventilation
PaO₂/FiO₂	= Ratio of arterial oxygen partial pressure to fraction of inspired oxygen
ROC	= Receiver operating characteristic
SAH	= Systemic arterial hypertension
SAPS	= Simplified Acute Physiology Score
SD	= Standard deviation
SOFA	= Sequential Organ Failure Assessment
VAP	= Ventilator-associated pneumonia

Acute myocardial infarction (AMI) is the leading cause of death in Brazil and worldwide. In 2017, according to the information technology department of the Brazilian Sistema Único de Saúde (also known as DATASUS), 7.06% (92,657 patients) of the total number of deaths were caused by AMI. And approximately 5 to 10% of patients with acute coronary syndrome require coronary artery bypass grafting (CABG)^[1]^.

CABG is the most performed cardiac surgery in Brazil, covering 54.1% of surgical cases. Considered the standard treatment for coronary artery disease, its indication is well established and can provide symptomatic improvement and prevent ischemic complications^[2,3]^. Despite being a safe procedure, risk factors and possible perioperative and postoperative complications may affect the mortality rate related to cardiac surgery, differing according to each center^[4]^.

Possible postoperative complications are directly related to risk factors, including age, systemic arterial hypertension (SAH), diabetes mellitus (DM), obesity, and smoking; these lead to a higher risk of complications and death^[5]^. Studies reveal a high prevalence of postoperative complications after major procedures, with pulmonary complications being the predominant ones^[6,7]^. That’s because the procedure in question (*i.e.*, CABG) causes an inflammatory response that affects multiple organs as well as their functions^[8]^.

CABG is considered a major surgery, with intensive care needs in the postoperative period, and CABG patients are often admitted to the intensive care unit (ICU) for their recovery and can often evolve with one of the main and most common infections, the ventilator-associated pneumonia (VAP), generally associated with a significant increase in morbidity and mortality; in patients on mechanical ventilation (MV), especially those in prolonged use, there is an increased risk of developing it from 7 to 21%^[9]^. Patients submitted to this type of surgery often remain on MV for a long time, which may represent this high risk of developing VAP^[10,11]^.

VAP is related to several types of pathogens, with *Pseudomonas aeruginosa* being the most frequently detected bacterium (around 20% of all cases), in addition to other types such as *Staphylococcus aureus, Klebsiella, Acinetobacter*, etc^[10]^. This contamination usually occurs during the process of intubation or aspiration of the secretion around the endotracheal tube and combined with the systemic inflammatory reaction that occurs in cardiac surgery, in addition to other factors, this nosocomial infection is found^[11]^.

The use of risk scales represents a great tool to estimate the results and the necessary medical efforts, being able to predict and calculate the possible postoperative complications^[12]^. Although they use different criteria, indices contribute significantly to the assessment of patients, as they predict organ dysfunction as well as mortality risk^[13,14]^.

Therefore, knowing the clinical profile of patients and the outcomes related to mortality, in addition to the complications resulting from the procedure, can provide information and ease the development of more individualized plans, aiming to reduce postoperative complications^[15,16]^.

This article aimed to describe clinical and demographic factors related to mortality of patients from a single center who underwent CABG and invasive MV between 2013 and 2019.

## METHODS

A retrospective study was carried out analyzing the data found in the Hospital de Clínicas da Universidade Estadual de Campinas database in the city of Campinas (São Paulo, Brazil) of patients who underwent cardiac surgery and who remained in the adult ICU after the procedure between 2013 and 2019. A convenience sample was used. There were no exclusions of patients after applying eligibility criteria. Eligibility criteria were restrictive, allowing only the inclusion of surgical patients. All patients undergoing cardiac surgery during this period were selected. An analysis was performed to verify which of these patients developed VAP in the postoperative period.

Eligibility criteria for inclusion in the study were patients aged 18 years or older and admitted to a surgical ICU for postoperative recovery from an elective or urgent surgical procedure. Among the patients who underwent cardiac surgery, only those who underwent CABG were included in this second moment and were separated into two different groups. Those with a diagnosis of VAP were included in Group 1 (VAP). To fit this diagnosis, the criteria of the Center for Control of Hospital Infections of the Hospital de Clínicas da Universidade Estadual de Campinas were followed, which included patients who presented a new or progressive pulmonary infiltration in radiographic examination of the lung, associated with two or more of the symptoms - fever (> 38.5°C) or hypothermia (< 36°C), leukocytosis (> 12 × 109 L), purulent tracheal secretion, or reduced oxygenation index (ratio of arterial oxygen partial pressure to fraction of inspired oxygen [PaO₂/FiO₂]) of ≥ 15% - in the previous 48 hours, in addition to having a positive bacterial culture. Based on these criteria, only patients classified as having VAP were included in Group 1 (VAP), that is, those who developed other types of complications were included in Group 2 (non-VAP).

In addition to the diagnosis of VAP and non-VAP, which separated the patients into different groups, data on the total incidence of myocardial revascularization in a total of patients undergoing cardiac surgery were analyzed. The incidence of VAP, an important postoperative complication in all patients after heart surgery, was also analyzed, as well as the death rate among patients who underwent myocardial revascularization and who progressed to VAP.

This study was approved by the Ethics Committee of the Universidade Estadual de Campinas (08905619.0.0000.5404) and statistical analysis was performed using the PASW Statistics 17 software (SPSS Inc., Chicago, Illinois, United States of America). Descriptive statistics were expressed as mean ± standard deviation and frequency. A *P*-value < 0.05 was considered statistically significant. This study was based on analysis of log data with outcomes and predictors available prior to initiating any form of statistical analysis. Therefore, it is a non-blinded study, where the results or predictors were not used.

## RESULTS

Data from 472 patients who underwent elective or emergency CABG between 2013 and 2019, with or without the use of cardiopulmonary bypass (extracorporeal circulation) during surgery, were analyzed. Among the individuals, 134 (28%) were women, and 338 (72%) were men. The mean age was 62.3 years, and the mean body mass index was 27.3. The patients’ hospital stay average was 10.3 days - 5.3 days in the ICU and 1.9 days on MV (Table 1).

**Table 1:** Characteristics of the participants of the study.

	Average	SD	Minimum	Maximum
Age (years)	62.3	9.4	33.0	87.0
Body mass (kg)	74.8	14.3	39.0	135.0
BMI (kg/m^2^)	27.3	4.8	16.5	47.8
ICU days	5.3	8.1	1.0	91.0
Hospital days	10.1	14.0	1.0	115.0
MV days	1.9	5.1	1.0	67.0

BMI=body mass index; ICU=intensive care unit; MV=mechanical ventilation; SD=standard deviation

**Fig. 1:**
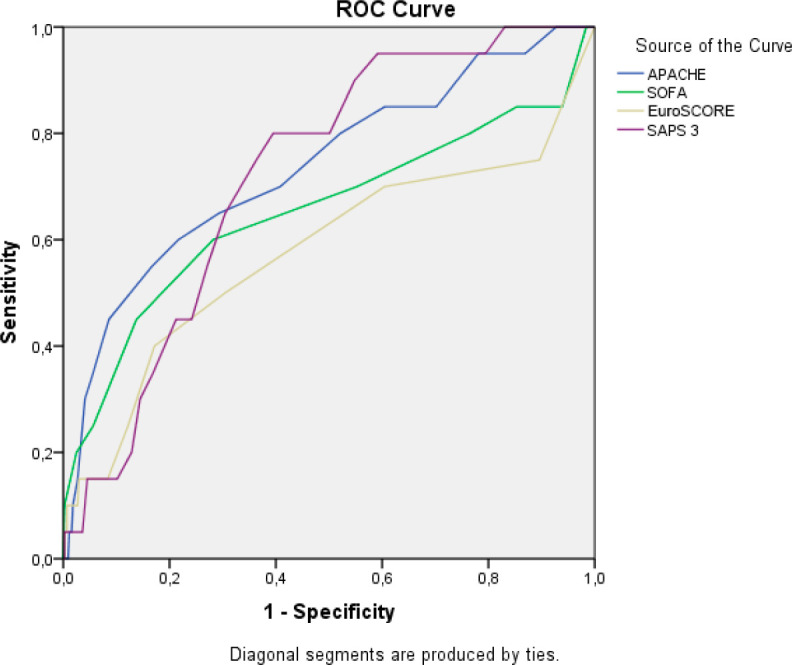
ROC curve and table of prognostic indices and mortality.

**Fig. 2:**
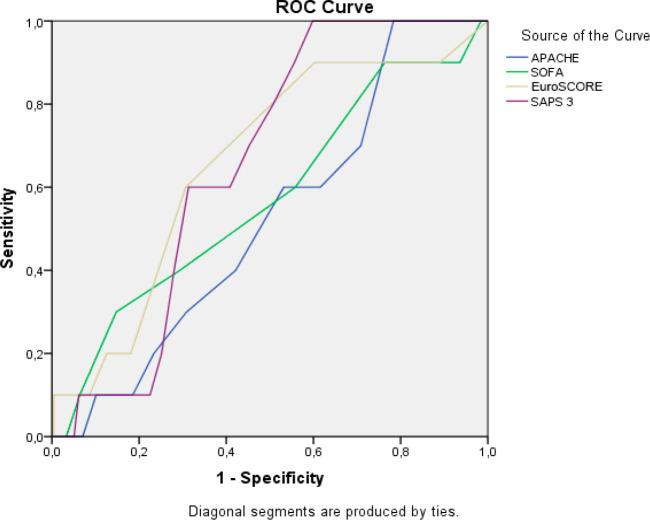
ROC curve and table of prognostic indices and VAP.

Concerning the characteristics and comorbidities associated with the study participants, a high frequency of SAH and a low frequency of alcoholics were found. A low frequency of VAP, death, and hospital death in addition to a low incidence of intercurrence therapy - indicating low frequency of clinical complications - were observed, with hemotherapy being the most frequent treatment (Table 2). Table 3 presents the Acute Physiology and Chronic Health Evaluation (APACHE), Sequential Organ Failure Assessment (SOFA), and Simplified Acute Physiology Score (SAPS) 3 characteristics of the analyzed patients. Table 4 presents the association between death and other qualitative variables of the study and shows that there was a significant association between VAP and death, revealing a higher frequency of death in positive VAP situations. A significant association was also found between hemotherapy and hemodialysis with death, revealing a higher prevalence of both in death situations. Table 5 shows the association between death and other quantitative variables in the study. It was found that at death, patients had statistically higher values for age, height, ICU days, APACHE, SOFA, and SAPS 3. Table 6 shows the association between VAP and other quantitative variables in the study. It is verified that in the presence of VAP, patients present statistically longer length of stay in the ICU and hospital. Figure 1 presents the receiver operating characteristic (ROC) curve of the prognostic and mortality indices. We found that APACHE and SAPS 3 present significant values related to mortality. Adequate area under the curve (> 70%) also reveals a high probability of correctly classifying patients. This presents the cutoff point that would indicate death for each of the prognostic indices with their respective sensitivity and specificity. Figure 2 presents the curve and ROC table of the prognostic indices and VAP. We verified that no index presented significant values.

**Table 2:** Characteristics of the participants of the study and frequency of VAP, death, and in-hospital death.

Variable	Presence	Absence	Total
SAH	381 (81%)	89 (19%)	470 (100%)
DM	216 (46%)	254 (54%)	470 (100%)
Alcoholic	54 (11%)	416 (89%)	470 (100%)
Active smoker	231 (49%)	239 (51%)	470 (100%)
VAP	10 (2%)	462 (98%)	472 (100%)
Death	20 (4%)	452 (96%)	472 (100%)
Hospital death	11 (2%)	461 (98%)	472 (100%)
Hemotherapy	68 (14%)	404 (86%)	472 (100%)
Hemodialysis	24 (5%)	448 (95%)	472 (100%)
Tracheostomy	5 (1%)	467 (99%)	472 (100%)

DM=diabetes mellitus; SAH=systemic arterial hypertension; VAP=ventilator-associated pneumonia

**Table 3:** Characteristics of APACHE, SOFA, EuroSCORE and SAPS 3.

	Average	SD	Minimum	Maximum
APACHE	12.9	4.1	3.0	27.0
SOFA	5.6	1.9	1.0	11.0
EuroSCORE	2.5	3.1	0.0	31.0
SAPS 3	37.3	8.0	3.0	68.0

APACHE=Acute Physiology and Chronic Health Evaluation; EuroSCORE=European System for Cardiac Operative Risk Evaluation; SAPS=Simplified Acute Physiology Score; SD=standard deviation; SOFA=Sequential Organ Failure Assessment

**Table 4:** Association between death and other qualitative variables of the study.

		Death			
Variable		Absent	Present	Total	Chi-square test
VAP	Absent	447 (97%)	15 (3%)	462 (100%)	χ^2^=52.728
	Present	5 (50%)	5 (50%)	10 (100%)	*P*<0.001^*^
SAH	Absent	86 (97%)	3 (3%)	89 (100%)	χ^2^=0.211
	Present	364 (95%)	17 (5%)	381 (100%)	*P*=0.646
DM	Absent	245 (96%)	9 (4%)	254 (100%)	χ^2^=0.688
	Present	205 (95%)	11 (5%)	216 (100%)	*P*=0.407
Alcoholic	Absent	397 (95%)	19 (5%)	416 (100%)	χ^2^=0.865
	Present	53 (98%)	1 (2%)	54 (100%)	*P*=0.352
Active smoker	Absent	229 (96%)	10 (4%)	239 (100%)	χ^2^=0.006
	Present	221 (96%)	10 (4%)	231 (100%)	*P*=0.938
Hemotherapy	Absent	397 (98%)	7 (2%)	404 (100%)	χ^2^=43.352
	Present	55 (81%)	13 (20%)	68 (100%)	*P*<0.001^*^
Hemodialysis	Absent	436 (97%)	12 (3%)	448 (100%)	χ^2^=52.754
	Present	16 (67%)	8 (33%)	24 (100%)	*P*<0.001^*^
Tracheostomy	Absent	448 (96%)	19 (4%)	467 (100%)	χ^2^=3.094
	Present	4 (80%)	1 (20%)	5 (100%)	*P*=0.079

DM=diabetes mellitus; SAH=systemic arterial hypertension; VAP=ventilator-associated pneumonia ^*^Statistically significant difference in the Mann-Whitney U test (*P*<0.05)

**Table 5:** Association between death and other quantitative variables in the study.

	Death		
Variable	Absent	Present	*P*-value
Age (years)	62.0 (9.4)	68.1 (7.5)	0.003^*^
Body mass (kg)	74.8 (14.2)	74.6 (8.9)	0.746
BMI	21.2 (4.7)	29.0 (5.9)	0.243
ICU days	4.7 (5.6)	18.0 (26.0)	< 0.001^*^
Hospital days	9.7 (13.2)	18.0 (26.0)	0.104
APACHE	12.8 (3.4)	16.8 (4.8)	< 0.001^*^
SOFA	5.6 (1.8)	6.9 (2.8)	0.016^*^
EuroSCORE	2.4 (2.7)	4.5 (6.7)	0.230
SAPS 3	37.0 (7.9)	43.2 (7.6)	0.001^*^

APACHE=Acute Physiology and Chronic Health Evaluation; BMI=body mass index; EuroSCORE=European System for Cardiac Operative Risk Evaluation; ICU=intensive care unit; SAPS=Simplified Acute Physiology Score; SOFA=Sequential Organ Failure Assessment Data presented as an average (standard deviation) ^*^Statistically significant difference in the Mann-Whitney U test (*P*<0.05)

**Table 6:** Association between VAP and other quantitative variables of the study.

	VAP		
Variable	No	Yes	*P*-value
Age (years)	62.2 (9.4)	65.4 (10.0)	0.307
Body mass (kg)	74.8 (14.3)	73.7 (14.7)	0.628
Height (cm)	165.6 (8.9)	163.4 (9.8)	0.643
BMI (kg/m^2^)	27.2 (4.7)	27.8 (7.0)	0.902
ICU days	4.8 (6.8)	26.2 (22.8)	< 0.001^*^
Hospital days	9.5 (12.9)	36.6 (30.6)	< 0.001^*^
APACHE	12.9 (4.1)	13.1 (2.9)	0.765
SOFA	5.6 (1.9)	6.1 (2.0)	0.419
EuroSCORE	2.5 (3.0)	4.2 (5.3)	0.073
SAPS 3	37.3 (8.0)	40.6 (4.6)	0.091

APACHE=Acute Physiology and Chronic Health Evaluation; BMI=body mass index; EuroSCORE=European System for Cardiac Operative Risk Evaluation; ICU=intensive care unit; SAPS=Simplified Acute Physiology Score; SOFA=Sequential Organ Failure Assessment; VAP=ventilator-associated pneumonia Data presented as an average (standard deviation) ^*^Statistically significant difference in the Mann-Whitney test (*P*<0.05)

**Table:** 

Indices	Area	Sensitivity	Specificity	Criterion	*P*-value
APACHE	0.737	60.0	78.7	> 15	< 0.001
SAPS 3	0.721	80.0	60.5	> 38	< 0.001
SOFA	0.653	60.0	71.2	> 6	0.020
EuroSCORE	0.577	40.0	82.7	> 3	0.247

APACHE=Acute Physiology and Chronic Health Evaluation; EuroSCORE=European System for Cardiac Operative Risk Evaluation; ROC=receiver operating characteristic; SAPS=Simplified Acute Physiology Score; SOFA=Sequential Organ Failure Assessment

**Table:** 

Indices	Area	Sensitivity	Specificity	Criterion	*P*-value
APACHE	0.521	100.0	22.5	> 9	0.818
SAPS 3	0.656	100.0	40.2	> 34	0.091
SOFA	0.572	30.0	85.4	> 7	0.434
EuroSCORE	0.660	90.0	39.9	> 1	0.082

APACHE=Acute Physiology and Chronic Health Evaluation; EuroSCORE=European System for Cardiac Operative Risk Evaluation; ROC=receiver operating characteristic; SAPS=Simplified Acute Physiology Score; SOFA=Sequential Organ Failure Assessment; VAP=ventilator-associated pneumonia

## DISCUSSION

In this retrospective study, data from 472 patients of a single center who underwent CABG were analyzed. The proposal was to identify variables such as risk factors and clinical data related to patients (*e.g.*, length of stay and MV) and to investigate their relationship with mortality rate. Complications in the postoperative period were also analyzed, such as VAP, use of hemotherapics, and hemodialysis in order to relate to a longer hospital stay, longer ICU time, and, consequently, an increase in the mortality rate.

Currently, CABG is still considered the gold standard in the treatment of patients with multivessel coronary disease, aiming to improve the quality of life and increase survival in patients. However, the surgical results are closely related to the clinical conditions of the patient, who most often have cardiovascular risk factors and associated comorbidities^[17]^.

Regarding mortality in cardiac surgeries, there is a great evolution, which depends on the number of surgeries performed and the type of procedure. On average, it varies from 0.7% in North Americans, while the same procedure can reach 20.8% in some Brazilian centers^[18]^. Vogt et al.^[19]^ observed, in a multicenter study, that mortality rates in different types of cardiac surgery ranged from 0.9% to 10.7%^[20]^. In this study, mortality was 4%, a rate relatively close to the study carried out in 2010 on the incidence of post-mortem pulmonary complications, where the mortality rate was 5.4%.

Several factors may contribute to higher mortality. Among the variables analyzed in the present study, the age of the patients who underwent surgery was, on average, 62 years. The increase in life expectancy shows an increase in the number of elderly people undergoing cardiac surgery, and advanced age shows a proportional relationship to the complications resulting from the surgical process as well as the time of MV^[21]^. The elderly generally have a different risk profile than younger people. The elderly have a higher prevalence of comorbidities such as SAH and DM^[22]^, and this higher prevalence of cardiovascular risk factors among patients undergoing CABG seems to be responsible for the increase in postoperative mortality in this population^[23]^.

In Brazil, patients referred for CABG are most often those with SAH and DM. These cardiovascular risk factors, as well as smoking, appear to be highly related to mortality^[17]^. In the present study, SAH and DM comorbidities were present in 81% and 46% of the patients, respectively. Recent studies have shown that the presence of DM is an independent risk factor for late postoperative CABG mortality, with a probability of death from cardiac causes being 1.73%, and 2.94% for overall mortality^[24]^. In another study, a comparison of cardiovascular risk factors was performed between Brazilian patients and patients from developed countries and indicated a prevalence of SAH (90.7% *vs.* 60%), previous AMI (23.5% *vs.* 2%), and DM (37.2% *vs.* 29%) clearly higher in Brazil^[21]^.

According to some studies, the advancement of surgical techniques and resources have brought about a decrease in the occurrence of postoperative complications in patients undergoing cardiac surgery, however, they still exist and impact the mortality of these patients^[25]^. They occur in the perioperative period or up to 30 days later, altering the patient’s clinical condition, despite care during the procedure, leading to an increase in the mortality of patients undergoing surgical procedures^[26]^.

Among the most common complications, pulmonary complications are very often found in the later period, mainly are directly associated with risk factors as pre-existing comorbidities, and most of them contribute to a longer length of hospitalization and in the ICU, increasing mortality^[27]^.

A longer stay in the ICU is associated with a longer time on MV, which is usually used in the treatment of respiratory failure in the postoperative period. In this study, patients who died stayed longer in the ICU and, consequently, on MV compared to other patients. This relationship was also found in a study that showed that a longer length of stay in the ICU and hospital usually occurs due to clinical complications in the postoperative period^[13]^.

Several factors seem to be associated with longer ICU and hospital stays. The surgical procedure itself, in which there are inflammatory responses, combined with anesthesia, changes in lung function, and a longer time to weaning from MV, all of these contribute to a longer permanence, which can cause an increased risk of infections and a consequent increase in mortality. Oliveira et al.^[21]^ performed a study showing that a high rate of risk factors resulted in an increase in hospital stay (12.7 days).

VAP is one of the most frequent nosocomial infections among MV patients in the ICU. The prevalence of VAP was 2% in this study, a rate considered relatively low in relation to another study, where the incidence was high, ranging from 6% to 52%, depending on the population studied, and which demonstrates that the risk of VAP occurrence grows with each day of stay on MV^[11]^.

VAP is defined as an infection with the presence of pulmonary infiltrates on the chest X-ray, which may be associated with fever or hypothermia, leukocytosis, purulent pulmonary secretion, or reduced oxygenation index (PaO₂/FiO₂). It is the most frequent nosocomial infection and is generally related to a significant increase in morbidity and mortality, in addition to increasing costs due to longer hospital stay. Considered difficult to diagnose in critically ill patients, however, it is an important predictor of mortality, especially when caused by resistant microorganisms. Nevertheless, a good prognosis is attributed when treatment is started with early appropriate antibiotic regimen, preventing prolonged time on MV and reducing mortality^[10]^. Studies show that the best strategy is the prevention of VAP, since it is closely related to the number of deaths because it worsens the condition of patients who are on MV by increasing the length of stay. Measures such as early extubation, strict hand hygiene, patient oral care, cuff management, and early administration of antibiotic therapy should be taken^[11]^.

The admission of surgical patients to the ICU is common in the postoperative period and is associated with monitoring and procedures. Information about the clinical condition and the risks of complications is necessary to follow the evolution and therapeutic results^[12]^. Some prognostic indices were developed to measure the severity of patients admitted to the ICU, in order to assess the performance of the ICUs and the therapeutic strategies used. The indices show, numerically, the probability of mortality^[13,14]^.

The mortality risk scales (SAPS 3 and APACHE II) used in this study calculate the severity of the patient using variables. An organ dysfunction measurement scale (SOFA) and a cardiac surgery risk scale (European System for Cardiac Operative Risk Evaluation, also known as EuroSCORE) were also used. The results of this study show that the SAPS 3 and APACHE II indices had a significant relationship with the mortality rate.

The performance of the prognostic index may differ when applied to different populations^[28]^. They are used in many studies in Brazil and worldwide, and some elderly patients may consider them useful in the ICU, as they show a response to the need for treatment of critically ill patients and intensive care patients^[29]^. Although these indices are widely used for comparative estimates, some studies show a better performance in low-risk patients or in comparison with high-risk patients^[12]^.

Finally, in addition to mortality-related factors, the ROC curve was used to demonstrate the sensitivity and probability of a true positive result. In this analysis, it is possible to verify significant values related to mortality with an adequate area under the curve with a high probability of correct classification of patients where the cutoff point indicates death in each of the prognostic indices, showing that APACHE and SAPS 3 present significant values related to mortality. These data confirm the results in the study by Falcão et al.^[12]^, which concluded that the results of the scores are tools that help in the prediction of mortality.

Although all the risk factors are considerable, in this casuistry, it was evidenced that of the selected patients, the death rate was higher in patients with older age, associated comorbidities, and those who spent longer time in the ICU and on MV.

## CONCLUSION

The present study showed that, in cardiac surgery patients, the variables age, duration of MV, and risk score are significantly associated with a higher mortality rate. Those with longer ICU stays, VAP, and higher risk scores also had higher mortality. However, the implementation and continuous use of a database, which includes information on surgical and postoperative procedures, can help the therapeutic routine. However, further studies are necessary, using different populations for this, aiming at the association of risk factors and postoperative complications with the mortality rate.
